# Progressive Decrease in Function and Ambulation Potential: A Case of Subacute Combined Degeneration

**DOI:** 10.7759/cureus.64027

**Published:** 2024-07-07

**Authors:** Andrew W Demko, David H Rustom

**Affiliations:** 1 Pain Management, Wayne State University Detroit Medical Center, Detroit, USA

**Keywords:** degenerative spinal disease, scd, pain medicine, vitamin b12 insufficiency, subacute combined degeneration

## Abstract

Subacute combined degeneration (SCD) is a reversible cause of posterior and lateral spinal cord degeneration. Prolonged vitamin B12 deficiency is a common cause of SCD as it leads to inhibition of proper myelin synthesis and reduces myelin integrity. When left untreated, SCD causes progressive debility that can lead to irreversible damage. We describe the case of a 49-year-old male patient who presented with one year of worsening weakness, back pain, paresthesias, and gait abnormalities. Laboratory values revealed vitamin B12 deficiency, elevated homocysteine and methylmalonic acid, and megaloblastic anemia. Following a diagnosis of SCD, the patient began treatment with intramuscular vitamin B12, and his pain and ambulation improved considerably in the following weeks. Prompt identification of vitamin B12 deficiency can lead to considerable improvements in function and quality of life.

## Introduction

Subacute combined degeneration (SCD) is a degenerative disease affecting the myelination of the posterior and lateral columns of the spinal cord. Patients may have motor, sensory, and cognitive deficits [1]. Vitamin B12 deficiency is a common cause of SCD due to its role in myelin synthesis and integrity, and a buildup of metabolites caused by vitamin B12 deficiency inhibits these processes [2,3]. Vitamin B12 deficiency is commonly associated with vegan diets and alcoholism, but other causes include autoimmune conditions such as pernicious anemia, nitrous oxide abuse, and malabsorption following gastrectomy [4]. Not all patients with vitamin B12 deficiency will have SCD, and it is generally seen in the middle-aged and elderly. Recently, the incidence of SCD in young adults has increased due to nitrous oxide abuse [1,4].

The diagnostic workup for SCD involves a combination of physical exam findings, diagnostic imaging, and laboratory analysis. Common exam findings include ataxia, weakness, and sensory disturbances [1,4]. MRI may be used to diagnose SCD, but it is normal in many patients with the condition [5]. Therefore, a laboratory workup is particularly important to identify the root causes of SCD, including vitamin B12 levels, copper and zinc levels, and biomarkers sensitive to insufficient vitamin B12, such as methylmalonic acid [1,3]. SCD causes progressively worsening demyelination and can result in permanent damage if left untreated, but most patients with the condition make a full recovery with vitamin B12 replacement [6].

Here, we will present a case of SCD due to vitamin B12 deficiency in the absence of dietary restrictions or drug abuse. The patient’s progressive weakness, difficulty walking, and back pain led to a diagnosis of SCD once he was found to have a severe vitamin B12 deficiency. This patient had a uniquely severe vitamin B12 deficiency compared to other outpatient examples in the literature, but still responded to treatment with intramuscular vitamin B12 replacement.

## Case presentation

A 49-year-old man was evaluated for one year of progressive back pain and paresthesias. He described a sharp, stabbing back pain in the thoracic region that radiated to the abdomen and both legs, greatly affecting mobility. During the past year, he had seen several other providers for his pain without resolution. The pain and mobility deficits were ameliorated by gabapentin and nonsteroidal anti-inflammatory drugs. Physical therapy prior to his outpatient visit was ineffective. He denied any alcohol or substance use. His decreased mobility kept him from being able to work for the past two months. During this time, he also acquired chronic constipation and generalized abdominal pain. On examination, he was alert and oriented. He was found to have distal lower extremity weakness, proprioceptive deficits, and reduced sensation. Both lower extremities had an increased tone, which was most pronounced on the left.

Blood tests showed a serum vitamin B12 level below the lowest detectable value of 50 pg/ml. Folate levels were within normal limits, but homocysteine was severely elevated at >132 μmol/L, and methylmalonic acid was elevated at 68.3 μmol/L. Anemia was detected with a hemoglobin of 10.2 g/dL and a hematocrit of 32.3%, and the mean corpuscular volume was above normal limits at 120.1 fL. Copper and zinc levels were within normal limits at 112 μg/dl and 108 μg/dl. TSH levels were also within normal limits. An autoimmune workup was negative for ANCA, anti-dsDNA, anti-smooth muscle, anti-RNP, anti-SSA and SSB, anti-SCL-70, and anti-JO1 antibodies. Anti-MPO and serine protease 3 antibodies were also both found to be within normal limits.

The patient’s final diagnosis, SCD, was confirmed by his low vitamin B12 levels, bilateral balance deficits, weakness, and loss of proprioception. Other unique findings included his major elevations in homocysteine and methylmalonic acid. His condition and ambulation improved considerably following a series of weekly vitamin B12 injections.

## Discussion

**Figure 1:**
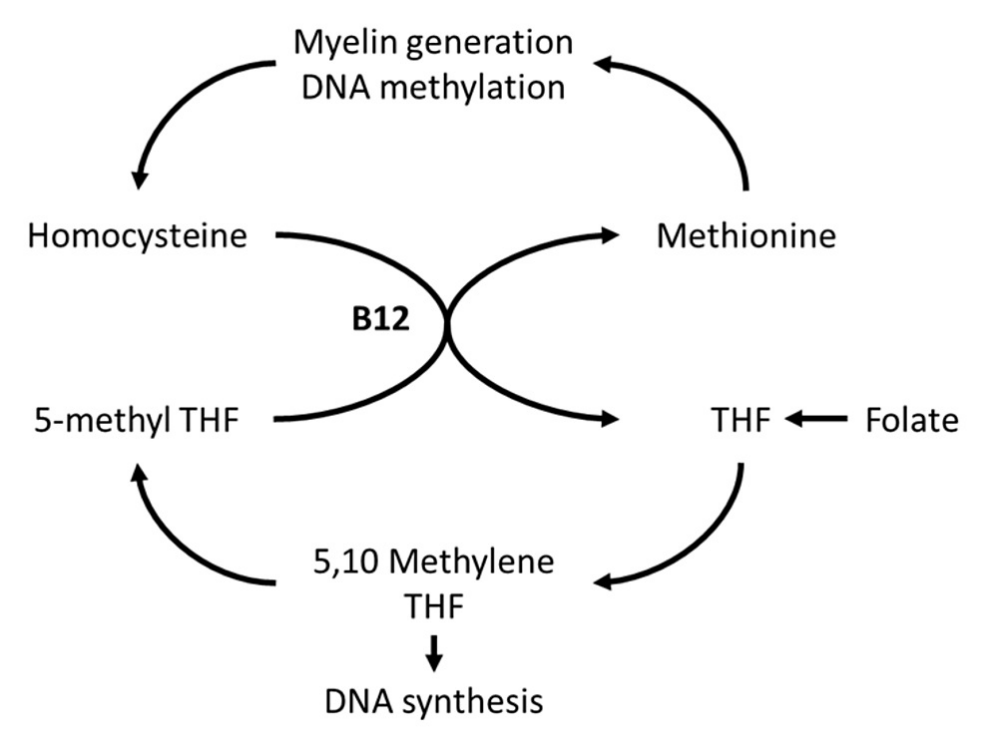
The cytoplasmic role of vitamin B12 in the folate and methionine cycles Biochemical reactions utilizing vitamin B12 as a cofactor are important for DNA synthesis and myelin generation [2,3]. THF, tetrahydrofolate Image credit: Andrew W. Demko

**Figure 2:**
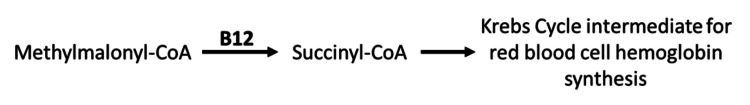
The mitochondrial role of vitamin B12 in energy generation Vitamin B12 is a cofactor for the conversion of methylmalonyl-coenzyme A to succinyl-coenzyme A [3,7]. Image credit: Andrew W. Demko

Vitamin B12 is a cofactor for many methyltransferase reactions important for synthesis and overall function. Vitamin B12 deficiency inhibits myelin synthesis and reduces lipid integrity, leading to the degenerative demyelination seen in SCD [2,3]. Vitamin B12 is essential for methionine synthesis, a reaction connecting the folate and methionine cycles (Figure 1). Products of these cycles are essential for DNA synthesis and myelin generation, and a lack of vitamin B12 leads to an improper buildup of metabolites that inhibit these processes [1-3]. In mitochondria, vitamin B12 serves as a cofactor for succinyl-CoA synthesis, an essential source of energy in the Krebs cycle of developing erythrocytes (Figure 2). Vitamin B12 deficiency can result from malnutrition, alcoholism, autoimmune conditions, nitrous oxide abuse, copper deficiency, and a lack of absorption, as seen in postoperative gastrectomy [1,4].

Patients presenting with SCD typically feature symptoms of ataxia, back pain, paresthesias, symmetric weakness, bilateral hyperreflexia, and impaired vibratory sense. The most common preceding symptom can be stiffness. This is typically followed by progressive ataxia, pain, and weakness [4,7,8]. Peripheral neuropathy commonly copresents with SCD, and the presence of hyporeflexia in some extremities suggestive of peripheral neuropathy should not rule out a diagnosis of SCD. Studies have also shown increased tone and spasticity in patients with SCD, as demonstrated by our patient [9]. Electromyography and nerve conduction studies have been inconclusive, with peripheral nerve damage detected in 55% of patients [9]. Feelings of numbness and coldness, along with paresthesias, are also common [10]. Furthermore, cognitive deficits in processing speed and executive function have been demonstrated in existing literature [5,11].

Common lab abnormalities in SCD include vitamin B12 deficiency, megaloblastic anemia, elevated methylmalonic acid, and elevated homocysteine, all seen in our patient [1]. Methylmalonic acid and homocysteine represent inappropriate buildups of metabolites caused by insufficient vitamin B12 to facilitate reactions [3]. Although homocysteine may also be elevated in folate deficiency, elevated methylmalonic acid is specific for vitamin B12 deficiency [1]. Copper and zinc levels are also checked. Copper deficiency is an uncommon cause of SCD, and high zinc levels lead to copper deficiency [7,8,12]. Additionally, folate levels are checked in patients with suspected SCD, as a deficiency of this vitamin can also cause anemia (especially if persistent after vitamin B12 repletion). Parietal cells are responsible for reabsorbing vitamin B12; hence, anti-parietal cell antibodies are tested to rule out autoimmune gastritis [4,10]. Our patient had abdominal pain and constipation, which may indicate an underlying gastritis, but his autoimmune workup was unremarkable. Abnormal TSH levels suggest an autoimmune disease coexisting with pernicious anemia. Patients with vitamin B12 deficiency caused by eating disorders should also be checked for deficiencies in fat-soluble vitamins [11]. The literature has reported SCD in patients who did not have anemia or abnormal vitamin B12 levels [7,8].

MRI can sometimes be used to assist with the diagnosis of SCD and may also evaluate other suspected conditions such as epidural abscess or transverse myelitis. MRIs of patients with SCD may demonstrate T2-weighted hyperintensities in regions of the dorsal or lateral columns, often in the cervical or upper thoracic spinal cord [5,8]. However, a lack of spinal cord hyperintensities, as seen in our patient, does not rule out the diagnosis of SCD. In a study of 54 patients with confirmed vitamin B12 deficiency, gait disturbance, ataxia, and other symptoms of SCD, hyperintense MRI findings were only observed in eight patients [5]. Furthermore, the presence of these findings was not associated with increased clinical severity or a change in prognosis, although the lesions did disappear with clinical resolution of symptoms. The lack of sensitivity in detecting SCD on conventional MRI highlights the importance of clinical history, physical exam, and laboratory workup in forming a diagnosis of SCD.

Intramuscular injections of cyanocobalamin or hydroxocobalamin are the standard of care for treating SCD. In patients requiring hospitalization, a typical injection pattern is five daily injections of 1,000 μg of cyanocobalamin, with subsequent injections spaced out to weekly or monthly dosages. These may be adjusted based on the functional status of the patient and the feasibility of the injection schedule [1,4,9,12]. The bioavailability of oral vitamin B12 can be limited, especially in postgastrectomy or autoimmune conditions. Four to eight weeks of vitamin B12 supplementation are typically required for noticeable improvement of symptoms. In some instances, lifelong supplementation is necessary [1,5]. However, patients with prolonged disease and a delay in accessing care may end up with permanent neurologic damage or only a mild response to treatment. Poor prognostic indicators include greater than seven affected spinal segments, progressive sensory involvement, poor postural support requiring assistive devices, and patients with malabsorption [6,7]. Positive prognostic indicators include age younger than 50 years and reduced time to diagnosis. Ambulation without an assistive device is associated with complete recovery in 90% of patients [6].

## Conclusions

Vitamin B12 deficiency is an uncommon condition that, when identified, can lead to considerable improvements in function and quality of life. SCD may be caused by vitamin B12 deficiency and result in impairments in myelin integrity. These defects lead to sensory symptoms and weakness. A thorough laboratory workup is essential for the proper diagnosis of SCD and insight into potential root causes. Patients generally respond well to treatment with intramuscular vitamin B12 injections, but patients with widespread spinal involvement and an extended delay in seeking treatment may experience permanent damage.
